# Vaccine Attitudes Among Adults in a Southern European Region: Survey from Pre- to Post-COVID-19

**DOI:** 10.3390/vaccines13121204

**Published:** 2025-11-28

**Authors:** Myrian Pichiule-Castañeda, Alicia Serrano-de-la-Cruz, María-Felícitas Domínguez-Berjón, Ana Gandarillas-Grande

**Affiliations:** 1 Sub-Directorate General for the Surveillance of Public Health, Directorate General of Public Health, Regional Ministry of Health of Madrid, 28002 Madrid, Spain; felicitas.dominguez@salud.madrid.org (M.-F.D.-B.); ana.gandarillas@salud.madrid.org (A.G.-G.); 2 Hospital Central de la Defensa Gómez Ulla, 28047 Madrid, Spain; aliserra@ucm.es

**Keywords:** vaccination, vaccine hesitancy, vaccine refusal, health inequities, socioeconomic factors, gender perspective, COVID-19, pandemic preparedness

## Abstract

Background: Vaccine hesitancy and refusal are growing public health challenges, reflecting individual decisions and social inequalities. The COVID-19 pandemic reshaped vaccine perceptions and may have amplified pre-existing differences. This study analyzed the evolution of vaccine hesitancy and refusal among adults in the Community of Madrid (Spain) between 2019 and 2024. We also explored the associated sociodemographic profiles. Methods: A retrospective observational study was conducted using data from the Non-communicable Disease Risk-Factor Surveillance System through a computer-assisted telephone interview (CATI) survey, targeting adults aged 18–64 years. Four waves (2019, 2020, 2021, 2024) were analyzed. Prevalence rates with 95% confidence intervals (95%CI) were calculated. The associations with sociodemographic variables (sex, age, country of birth, education, and employment status) were assessed using Poisson regression models to obtain crude and adjusted prevalence ratios (aPR). Results: A total of 7978 participants were included (49.1% men; mean age 41.97 years). Vaccine hesitancy increased from 3.8% (95% CI: 3.0–4.7) in 2019 to 18.5% (95% CI: 16.8–20.2) in 2024; vaccine refusal increased from 2.1% (95% CI: 1.6–2.8) to 8.0% (95% CI: 6.9–9.3). Vaccine hesitancy and refusal adjusted for socioeconomic variables increased in 2024 compared to 2019 (PRa: 5.04; 95% CI: 3.96–6.41 and aPR: 4.00; 95% CI: 2.86–5.59, respectively). Hesitancy was associated with female sex and middle age in 2019, to middle age in 2020, to intermediate education and migrant origin in 2021, and to education and migrant status in 2024. Vaccine refusal showed a similar pattern to that of vaccine hesitancy, highlighting the association with socioeconomic vulnerability. Conclusion: Between 2019 and 2024, vaccine hesitancy and refusal increased, and the association with socioeconomic vulnerability has also increased. Equity-based vaccination strategies are needed in order to strengthen institutional trust and reduce structural barriers to vaccine acceptance.

## 1. Introduction

Vaccination is one of the most effective and cost-efficient public health measures for preventing communicable diseases and reducing related morbidity and mortality. Vaccine hesitancy is defined by the World Health Organization as “the delay in acceptance or refusal of vaccines despite availability of vaccination services” [1]. Therefore, hesitancy indicates uncertainty or postponement despite eventual acceptance. Vaccine refusal, on the other hand, indicates a firm decision not to vaccinate. Both represent different but interrelated barriers to achieving optimal immunization coverage and disease control [2,3].

The COVID-19 pandemic posed an unprecedented challenge, not only due to its health, social, and economic consequences, but also because of the exceptional speed at which vaccines against SARS-CoV-2 were developed and deployed [4]. Although vaccination was essential to mitigating transmission and disease burden, widespread uncertainty, perceptions of potential adverse effects, and the massive circulation of misinformation and disinformation influenced public attitudes toward vaccination more broadly [5,6], even in countries with historically high coverage rates.

Although vaccine hesitancy varies by region, it has increased overall following the onset of the pandemic. Along these lines, some studies suggest that 26% of people in European countries were not willing or planning to vaccinate against COVID-19 [7]. Similarly, in countries such as Canada, which has one of the highest rates of vaccination against COVID-19, there has been around a 20% decrease in the administration of boosters between 2021 and 2023 [8].

Models of the determinants of vaccine hesitancy include contextual, individual, and social group influences, as well as vaccine- and vaccination-specific issues [9]. Evidence consistently shows that vaccine hesitancy is not randomly distributed across the population but is patterned along sociodemographic and socioeconomic lines. Some studies suggest that women, younger people, those with lower levels of education, and those experiencing greater economic deprivation are more likely to be hesitant about vaccines [9].

The national COVID-19 vaccination campaign in Spain began on 27 December 2020, marking the start of the country’s immunization effort against the pandemic. The Community of Madrid launched its vaccination program on the same day [10].

Research on the temporal dynamics of vaccine hesitancy remains limited, particularly regarding how social inequalities shape changes in public trust before, during, and after the COVID-19 pandemic. This study analyzed the evolution of vaccine hesitancy and refusal among adults in the Community of Madrid (Spain) between 2019 and 2024. We also explored the associated sociodemographic profile.

## 2. Materials and Methods

### 2.1. Study Design and Population

This was a retrospective observational study based on a health representative survey. The data were obtained from the Non-communicable Disease Risk-Factor Surveillance System, targeting the adult population (18–64 years) (SIVFRENT-A) in the Community of Madrid (Spain). The analysis included four survey waves corresponding to the years 2019, 2020, 2021, and 2024. Items related to vaccine hesitancy and negative attitudes were included as part of the rotating modules in these years. Consequently, our analysis was limited to the survey waves in which these items were collected.

According to the latest data available from the Spanish National Statistics Institute, the Community of Madrid is one of the most densely populated regions in Spain, with a total population of 7,009,268 inhabitants as of 1 January 2024. About 54.2% of the total population lives in the city of Madrid and around 40% in its metropolitan area, which includes 24 municipalities with more than 50,000 inhabitants (10 of them exceeding 100,000). Madrid is also characterized by marked social and demographic diversity: 20.9% of its residents were born outside Spain [11].

The sample frame was the database of individuals with a health card of the public health system in the Madrid Region. Annual sampling was stratified by sex, age group (18–29, 30–44, and 45–64 years), and geographic area (Madrid city, metropolitan area, and the rest of municipalities). Within each stratum, respondents were randomly selected. Participation was anonymous, and data were collected monthly, excluding August.

### 2.2. Survey Procedure

The data were collected through a telephone survey using the CATI system (Computer-Assisted Telephone Interviewing). The health survey questionnaire included a fixed module (monitoring physical activity, diet, anthropometry, tobacco and alcohol use, preventive practices, and accidents) and other rotating modules that changed according to information needs, where questions about vaccine attitudes were included.

### 2.3. Study Variables

The main variables were both binary categorical (yes/no): (a) Vaccine hesitancy was assessed by the question: “Have you ever been hesitant or had doubts about getting vaccinated or vaccinating someone under your care?”; (b) Vaccine refusal was assessed by the question: “Have you ever refused to get vaccinated or to vaccinate someone under your care, even when vaccination was recommended by the immunization schedule, chronic condition, or medical advice?”. These questions were derived from the operationalization of the concept as defined in the literature, similar to those used in other studies in Spain [12].

Given that hesitancy and refusal refer to distinct attitudinal and behavioral dimensions, and are measured through independent questions in SIVFRENT-A, they were analyzed as separate outcomes.

Secondary variables were: (a) Sex: male or female. (b) Age: categorized as 18–30, 31–44, and 45–64 years. (c) Survey year: 2019, 2020, 2021, or 2024. (d) Geographic area: the city of Madrid, the metropolitan area, and the rest. (e) Country of birth classification followed the Human Development Report 2023/2024, using an HDI > 0.8 as the threshold for high development: Spain, other high-development countries, and other low-development countries. (f) Employment status: employed, unemployed, or other (student, unpaid household work, retired/pensioner). (g) Educational level: basic or lower, intermediate, and higher. (h) Occupational social class from high to less advantaged: high (categories I–II), medium (III), and low (IV–V) [13].

### 2.4. Statistical Analysis

A descriptive analysis of the surveyed population was performed; ANOVA was used to compare quantitative variables and the chi-square test to compare categorical variables. Prevalence rates for the two main outcomes (vaccine hesitancy and refusal) were calculated with 95% confidence intervals (95% CI). We also evaluated the degree of overlap between the two main outcomes to better understand their empirical relationship. Adjusted prevalence ratios (aPR) were estimated using Poisson regression models with robust variance. Adjustment variables included sex, age, country of birth, employment status, and educational level. Social class is not included in the model as it showed a moderate correlation with educational level. We also analyzed a first-order interaction between sex and year of study.

A *p*-value < 0.05 was considered statistically significant. Stata 18.0 (StataCorp, College Station, TX, USA) was used for the statistical analysis.

## 3. Results

### 3.1. Characteristics of the Studied Population

The analysis included 7978 adults interviewed between 2019 and 2024, with a balanced distribution across the four years of study. Table 1 summarizes the demographic and socioeconomic characteristics of the study sample. The mean age of participants was 41.97 years (SD 12.55), with no significant differences across periods. Overall, 49.1% were men and 50.9% women, maintaining a similar sex distribution over time.

The distribution by age group and geographic area remained stable, although some changes in the sociodemographic composition were observed. The proportion of individuals with higher education increased from 38.5% in 2019 to 48.5% in 2024 (*p* < 0.001), as did the proportion of those engaged in employment (from 77.9% to 83.9%; *p* < 0.001). The proportion of individuals in higher social classes (I–II) increased from 30.9% in 2019 to 38.1% in 2024 (*p* < 0.001). Differences were also observed by country of birth (*p* = 0.027), with a slight decrease in migrants from highly developed countries and an increase in those from less developed countries. As the survey is based on a representative sample, these findings reflect the social changes occurring in the region.

### 3.2. Prevalence of Vaccine Hesitancy and Refusal

Vaccine hesitancy among adults in the Community of Madrid increased between 2019 and 2024. In the pre-pandemic period (2019), hesitancy prevalence was 3.8% (95% CI: 3.0–4.7), rising slightly during the pandemic years (4.8% in 2020 and 8.2% in 2021) and reaching its peak in 2024 at 18.5% (95% CI: 16.8–20.2).

**Figure 1:**
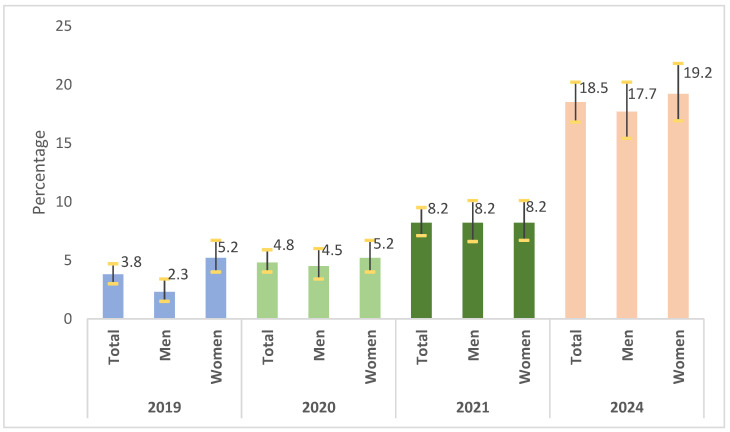
Vaccine hesitancy by year and by sex. Population aged 18–64 years. Community of Madrid (Spain), 2019–2024.

Sex-stratified analysis revealed that women showed slightly higher hesitancy rates than men before the pandemic (5.2% vs. 2.3% in 2019). These differences narrowed markedly in subsequent years, as an upward trend was observed for both groups, reaching 19.2% for women and 17.7% for men by 2024 (Figure 1).

**Figure 2:**
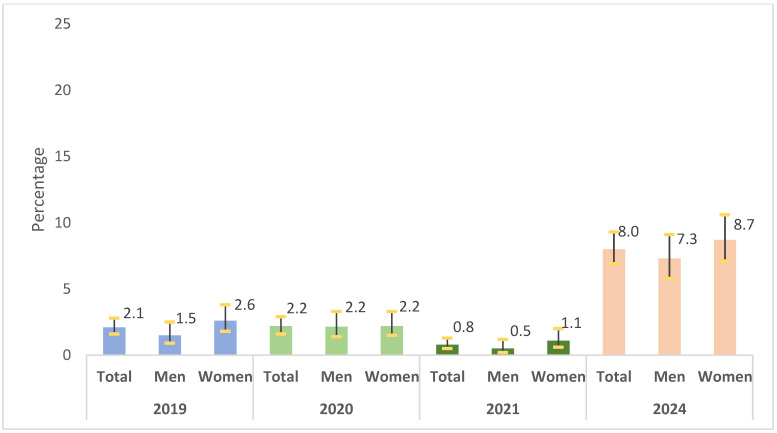
Vaccine refusal by year and sex. Population aged 18–64 years. Community of Madrid (Spain), 2019–2024.

Vaccine refusal remained lower and more stable than hesitancy throughout the pre- and mid-pandemic years, followed by a great increase in the post-pandemic period. Among adults overall, refusal rose from 2.1% in 2019 to 8.0% in 2024, with the lowest percentage recorded in 2021. Sex differences were small: refusal was slightly higher among women early on (2.6% vs. 1.5% in 2019), whereas by 2024 both sexes exhibited substantial increases, reaching 7.3% in men and 8.7% in women (Figure 2).

When analyzing the overlap between hesitancy and refusal, we found that 26.2% of individuals who expressed hesitancy also reported refusal, whereas refusal was extremely uncommon among those without hesitancy (1.1%). Conversely, 70.5% of participants who reported refusal also reported vaccine hesitancy, and most individuals who did not report vaccine refusal also did not report hesitancy (93.3%).

### 3.3. Adjusted Prevalence Ratio

Vaccine hesitancy and refusal adjusted for socioeconomic variables increased in 2024 compared to 2019 (aPR: 5.04; 95% CI: 3.96–6.41 and aPR: 4.00; 95% CI: 2.86–5.59, respectively) (Table 2). An interaction of the first order was found between year of study and sex with regard to vaccine hesitancy. Therefore, the analysis was sex-stratified. Vaccine hesitancy increased more among men than women, rising by aPR: 7.96; 95% CI: 5.15–12.31 and aPR: 3.80; 95% CI: 2.84–5.09, respectively, in 2024 compared to 2019.

### 3.4. Sex Differences

**Figure 3:**
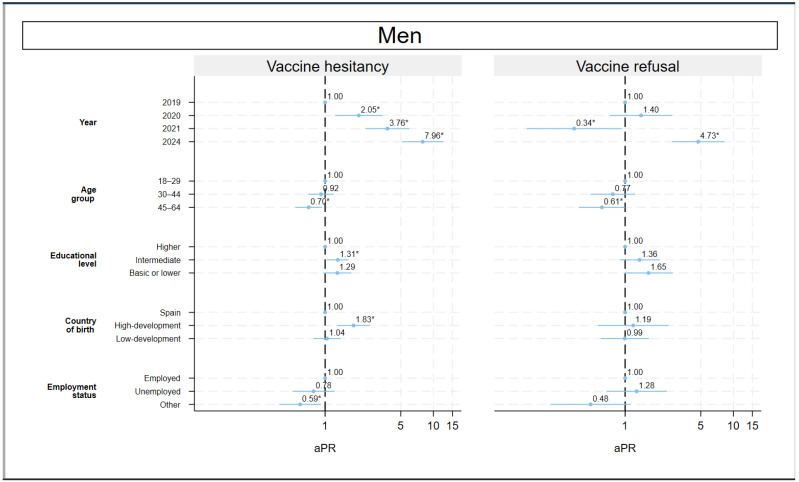

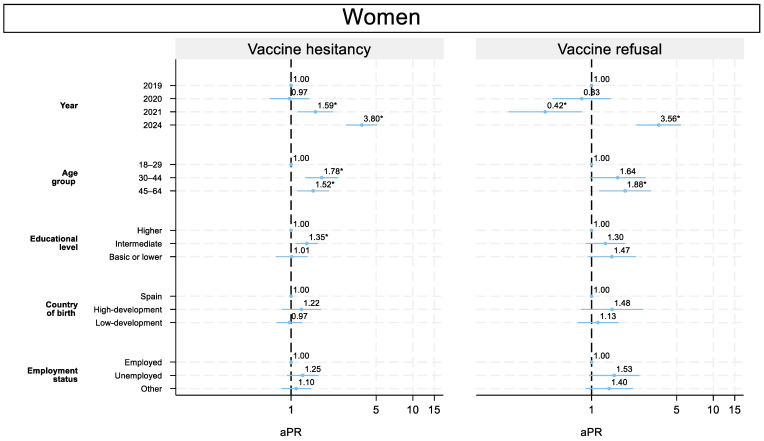
Adjusted prevalence ratio in vaccine hesitancy and refusal, sex-stratified. Population aged 18–64 years. Community of Madrid (Spain), 2019–2024. Note: aPR: adjusted prevalence ratio. * *p* < 0.05.

In the stratified analysis, differences were observed according to age group and employment status. Among women, older individuals were more hesitant (30–44 years old: aPR = 1.78; 95% CI: 1.32–2.42; 45–64 years old: aPR = 1.52; 95% CI: 1.13–2.04 compare with 18–29 years old), whereas younger individuals were more hesitant among men (45–64 years old: aPR = 0.70; 95% CI: 0.53–0.93 compare with 18–29 years old). A similar pattern emerges with regard to vaccine refusal (45–64 years old compared with 18–29 years old in women: aPR = 1.88; 95% CI: 1.16–3.07, and in men: aPR = 0.61; 95% CI: 0.37–1.00). According to employment status, greater hesitancy was observed among the unemployed women, while greater hesitancy was observed among employed men, although these differences were not statistically significant. In terms of educational level, it is worth noting that those with secondary education are more hesitant to be vaccinated than university graduates, among both men and women (aPR =1.31; 95% CI: 1.04–1.65 and aPR =1.35; 95% CI: 1.08–1.67, respectively). In men, there was also greater hesitancy in foreigners born in high-development countries (aPR =1.83; 95% CI: 1.30–2.59) compared to those born in Spain (Figure 3).

### 3.5. Evolution of the Association with the Socioeconomic Variables (2019–2024)

In 2019, hesitancy was driven by sex and age—particularly among middle-aged women—while refusal was rare and weakly patterned. In 2020, sex differences vanished, but age remained significant. In 2021, amid mass vaccination, education and migration became central, and a subtle shift emerged from female caution to a more balanced gender pattern. By 2024, hesitancy and refusal reached their highest prevalence, and the association with educationally and socioeconomically disadvantaged groups remained.

#### 3.5.1. 2019: Pre-COVID-19

**Figure 4:**
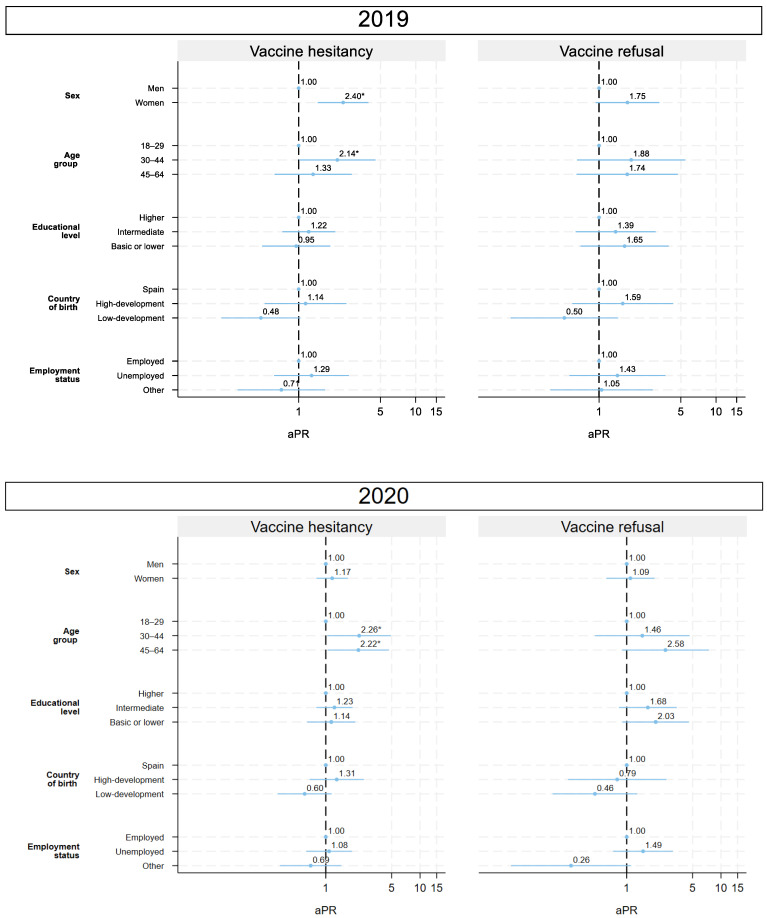
Adjusted prevalence ratio in vaccine hesitancy and refusal in 2019 and 2020. Population aged 18–64 years. Community of Madrid (Spain). Note: aPR: adjusted prevalence ratio. * *p* < 0.05.

In 2019, vaccine hesitancy in women was more than twice as common as in men (aPR = 2.40; 95% CI: 1.46–3.94). Age was also relevant: adults aged 30–44 years had higher hesitancy (aPR = 2.14; 95% CI: 1.01–4.53) compared with those aged 18–29 years; the sex-stratified analysis reveals that this association with age is only present among women. Unemployment showed a non-significant tendency towards greater hesitancy, whereas migrants born in low-development countries showed lower hesitancy. For vaccine refusal, there were non-significant trends toward higher prevalence among women, older adults, and those with a lower level of education (Figure 4).

#### 3.5.2. 2020: Onset of COVID-19

In 2020, the first year of the pandemic, patterns shifted slightly. No significant sex differences were observed. Age was still a determinant: adults aged 30–44 years (aPR = 2.26; 95% CI: 1.04–4.92) and 45–64 years (aPR = 2.22; 95% CI: 1.05–4.97) were more than twice as likely to show hesitancy compared with younger adults. The sex-stratified analysis reveals, again, that this association with age is only present among women. For vaccine refusal, there were non-significant trends toward higher prevalence among older adults and those with a lower level of education and unemployment (Figure 4).

#### 3.5.3. 2021: COVID-19 Vaccination Campaign

**Figure 5:**
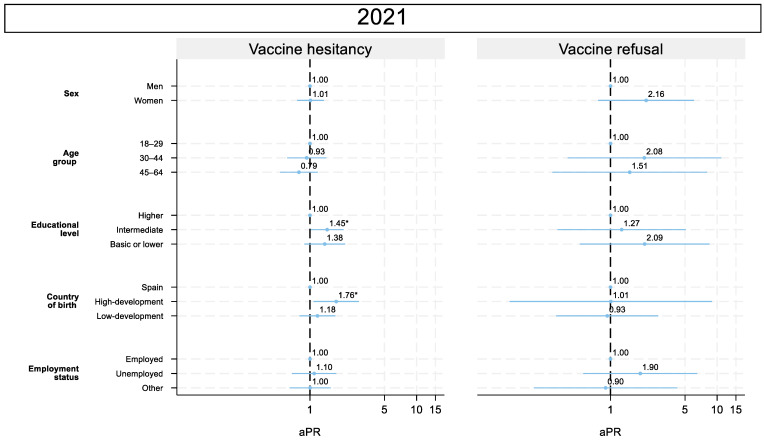

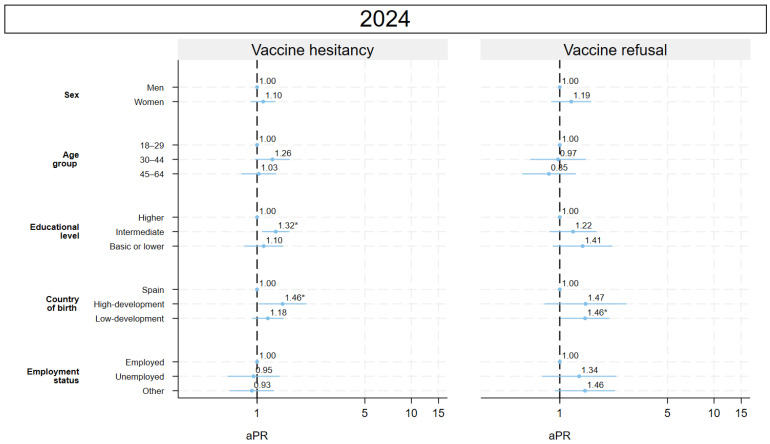
Adjusted prevalence ratio in vaccine hesitancy and refusal in 2020 and 2021. Population aged 18–64 years. Community of Madrid (Spain). Note: aPR: adjusted prevalence ratio. * *p* < 0.05.

In 2021, education and migration became relevant: those with intermediate education were more hesitant than those with a higher level of education (aPR = 1.45; 95% CI: 1.02–2.07). Participants born in highly developed countries showed greater hesitancy (aPR = 1.76; 95% CI: 1.08–2.88). The sex-stratified analysis reveals that in men, there is a significant trend toward more hesitancy in the younger population. For refusal, there were non-significant trends toward higher prevalence among women, adults aged 30–44 and 45–64 years, those with a lower level of education, and unemployed participants (Figure 5).

#### 3.5.4. 2024: Post-COVID-19

Socioeconomic disparities persisted in 2024. Those with an intermediate education level (aPR = 1.32; 95% CI: 1.08–1.62) and migrants born in highly developed countries (aPR = 1.46; 95% CI: 1.02–2.09) were more likely to be vaccine-hesitant. The sex-stratified analysis revealed a significant trend towards greater hesitancy among older women. For refusal, associated factors were migrants born in a low-development country (aPR = 1.46; 95% CI: 1.01–2.10), and neither sex nor age differences were found, although trends persisted in similar directions. The sex-stratified analysis reveals that in men, there is a significant trend of more refusal in the younger population. Also, there were non-significant trends towards a higher prevalence in those with a lower educational level and those who were unemployed (Figure 5).

## 4. Discussion

The results of this study show a substantial increase in vaccine hesitancy and refusal among the adult population in Madrid (Spain). These negative attitudes towards vaccination have increased more among men than women. The results also show a transformation in the associated factors with vaccine hesitancy and refusal. While before and during the pandemic, these differences were mainly explained by sex and age, in the post-pandemic period, unfavorable attitudes toward vaccination became associated with groups facing educational, migratory, and occupational disadvantages, reflecting a shift toward deeper social inequalities.

It is important to consider that vaccine hesitancy and vaccine refusal represent related but conceptually distinct constructs. Hesitancy reflects an attitudinal state of doubt or ambivalence, whereas refusal corresponds to a behavioral decision not to vaccinate despite medical or programmatic recommendations. Although both constructs lie on the same continuum of confidence, they do not fully overlap: individuals may express doubts yet ultimately accept vaccination, while refusal represents a narrower and more definitive behavioral subset.

Consistent with this conceptual differentiation, we examined the empirical overlap between hesitancy and refusal. As expected, refusal was mostly concentrated among individuals reporting hesitancy. Conversely, refusal was extremely uncommon among individuals without hesitancy. This pattern confirms that hesitancy represents a broader attitudinal construct, whereas refusal corresponds to a more restricted behavioral manifestation. Despite these differences, both outcomes displayed broadly similar sociodemographic gradients. For refusal, some associations did not reach statistical significance, a finding likely related to its lower prevalence and the concentration of events in specific subgroups rather than to substantively weaker associations.

These findings are consistent with trends observed in other research groups [7,8]. Some studies from 2022 indicate that around 7% of people refused to be vaccinated in 2022 [14]. Also, as per a cross-sectional study conducted in Spain in 2021, the vaccine refusal rates were 16.8% [15]. In our study, the temporary decline in refusal observed in 2021 may have been influenced by external requirements for proof of vaccination, which could reduce refusal independent of underlying attitudes. Although COVID-19 vaccination was not legally mandatory in Spain, proof of vaccination was routinely required to access workplaces, international travel, and many social and leisure activities. This created a ‘functionally mandatory’ environment that likely reduced behavioral refusal, even as attitudinal hesitancy continued to rise. In this context, refusal appears to have been constrained by structural incentives and social norms, whereas hesitancy remained more sensitive to perceptions of uncertainty, risk, and trust.

As in our study, other research has reported higher rates of vaccine hesitancy among women [16]. To properly assess these differences, it is necessary to take biological differences into account. The higher reactogenicity rate after the vaccination against the SARS-CoV-2 virus in women compared to men could influence vaccine hesitancy [17]. Also, the early gendered pattern of hesitancy, particularly among middle-aged women, could be interpreted not just as irrational distrust but rather as a reflection of gendered care responsibilities. The survey items referred not only to vaccination of oneself but also of dependents, suggesting that women—who more often assume caregiving roles—were evaluating vaccination risks and benefits for others under their care. This interpretation aligns with the literature describing women as health gatekeepers within families, showing both greater preventive engagement and heightened caution toward medical uncertainty.

The difference between men and women is striking in relation to age; it is noteworthy that, since 2021, there has been a tendency towards greater hesitancy and refusal among younger men. This may be related to young people having a lower perception of risk and great distrust toward new vaccines. Some studies have found that younger populations, especially those with lower levels of education, show greater resistance to vaccination [16]. In Spain, several post-COVID-19 studies have reported a general decline in vaccine confidence and an increase in polarization by educational level and socioeconomic position.

European studies suggest that, during and after the pandemic, distrust in vaccines was not only driven by individual concerns (such as side effects or uncertainty) but also became more common among socially vulnerable groups, indicating a shift toward institutional disaffection. In this context, the loss of trust in health institutions emerges as a structural factor rather than a simple individual perception. Vaccine hesitancy can be seen as an indicator of symbolic distance from health institutions, whereas vaccine refusal expresses a deeper rupture in the relationship of trust—one that is amplified by educational and economic inequalities. This temporal change suggests that vaccination campaigns succeeded in reducing initial uncertainty but failed to close the social gaps in trust toward the health system.

Overall, our models depict a clear transformation: from an early pattern shaped by care-related caution among women (2019–2020) to one characterized by institutional distrust and structural inequality (2021–2024). During the pandemic year (2021), vaccine refusal declined significantly in both sexes, whereas hesitancy continued to increase compared with the pre-pandemic period. This consistent pattern across sexes suggests that, while initial rejection declined during the vaccination campaign, doubts and ambivalence toward vaccination persisted and even expanded. Over time, vaccine attitudes moved from individual perceptions toward socially rooted disparities in trust and acceptance.

The disappearance of this gender gap in later years does not necessarily imply increased confidence among women but could instead indicate a relative rise in distrust among men, especially younger men. Recent social studies in Spain have documented growing institutional skepticism among young males, often linked to techno-libertarian or anti-statist discourses. In the vaccination domain, this mindset may translate into resistance toward public health recommendations perceived as symbols of state control.

Given this shift from individual concerns to structurally rooted distrust, reversing the current trend requires interventions that address both dimensions. Transparent communication about the benefits and risks of vaccination, together with dialogue-based counseling in primary care, can help respond to individual doubts and counter misinformation. At the structural level, targeted outreach to socially and economically vulnerable groups is essential, as vaccine attitudes increasingly reflect inequalities in institutional trust. Strengthening this trust also entails reducing administrative and informational barriers to vaccination and ensuring equitable access across population subgroups. In addition, coordinated, tailored public health messaging, community engagement, and continuous monitoring of misinformation have proven effective in improving vaccine acceptance in other European settings and may be crucial in reversing the trends observed in our study [18].

### Limitations of the Study

This study has several limitations that should be considered when interpreting the results. First, the items on vaccine hesitancy were developed through conceptual operationalization and are not based on a validated tool. They function as screening questions with high sensitivity, allowing trend analysis and helping to inform the incorporation of validated instruments in future health surveys. On the one hand, the variables analyzed refer to general vaccine hesitancy or refusal, without distinguishing between different types of vaccines (e.g., influenza, COVID-19, others), since vaccine acceptance can vary substantially depending on the disease, perceived risk, or prior experience with specific vaccination campaigns. On the other hand, these questions are directed both at the subjects themselves and at other people under their responsibility, so they would also be measuring certain gender issues. This lack of differentiation limits the precision and applicability of the findings. Also, items on vaccine hesitancy and refusal were not collected in 2022–2023 due to the rotating structure of SIVFRENT-A modules. This limits our ability to describe continuous year-to-year trends.

Second, the study used self-reported questionnaires as the main source of information. The SIVFRENT-A survey does not capture the specific motivations underlying hesitancy and refusal. Future cycles of SIVFRENT-A could benefit from incorporating items that explore these motivations in greater depth.

Third, the cross-sectional design used in this study only allows for the identification of associations between socioeconomic factors and attitudes toward vaccination, but it does not establish causal relationships.

Finally, the external validity of the results is limited by the geographical scope of the study, which is restricted to the Community of Madrid. Attitudes toward vaccination are influenced by social, cultural, political, and organizational factors that may differ substantially across other Spanish regions or countries. Consequently, the results cannot be directly extrapolated to different contexts.

## 5. Conclusions

Vaccine hesitancy and refusal increased between 2019 and 2024. Overall, the results reveal a transition from an initially diffuse, personal mistrust toward a structural inequality pattern in vaccine acceptance. This shift highlights the importance of addressing hesitancy and refusal from an equity perspective, integrating public health, education, and social cohesion policies.

In terms of surveillance, it is recommended to continue monitoring vaccine hesitancy and refusal as indicators of health inequity, not merely as individual behaviors.

## Figures and Tables

**Table 1:** Demographic and socioeconomic characteristics of the study population, by year (%). Population aged 18–64 years. Community of Madrid (Spain), 2019–2024.

Variable	2019 N (%)	2020 N (%)	2021 N (%)	2024 N (%)	Total N (%)	*p*-Value
N (% by row)	1997 (25.0)	2002 (25.1)	1990 (24.9)	1989 (24.9)	7978 (100.0)	
Mean age (years) (DS)	41.94 (12.50)	41.99 (12.51)	42.05 (12.83)	41.89 (12.38)	41.97 (12.55)	0.983
Age group						0.384
18–29 years	401 (20.1)	399 (19.9)	427 (21.5)	428 (21.5)	1655 (20.7)	
30–44 years	741 (37.1)	743 (37.1)	684 (34.4)	691 (34.7)	2859 (35.8)	
45–64 years	855 (42.8)	860 (43.0)	879 (44.2)	870 (43.7)	3464 (43.4)	
Sex						0.914
Men	974 (48.8)	975 (48.7)	978 (49.1)	989 (49.7)	3916 (49.1)	
Women	1023 (51.2)	1027 (51.3)	1012 (50.9)	1000 (50.3)	4062 (50.9)	
Country of birth						0.027
Spain	1533 (76.8)	1552 (77.5)	1569 (78.8)	1534 (77.1)	6188 (77.6)	
High-development country	138 (6.9)	137 (6.8)	108 (5.4)	99 (5.0)	482 (6.0)	
Low-development country	326 (16.3)	313 (15.6)	313 (15.7)	356 (17.9)	1308 (16.4)	
Geographic area						0.262
City of Madrid	970 (48.6)	972 (48.6)	975 (49.0)	971 (48.8)	3888 (48.7)	
Metropolitan area	824 (10.2)	826(41.3)	846(42.5)	852(42.8)	3348(42.0)	
Rest	203 (10.2)	204 (10.2)	169 (8.5)	166 (8.3)	742 (9.3)	
Educational level						<0.001
Higher	769 (38.5)	809 (40.5)	798 (40.1)	962 (48.5)	3338 (41.9)	
Intermediate	776 (38.9)	815 (40.8)	809 (40.7)	729 (36.8)	3129 (39.3)	
Basic or lower	452 (22.6)	376 (18.8)	383 (19.2)	292 (14.7)	1503 (18.9)	
Employment status						<0.001
Employed	1555 (77.9)	1456 (72.7)	1498 (75.3)	1669 (83.9)	6178 (77.4)	
Unemployed	157 (7.9)	265 (13.2)	188 (9.4)	122 (6.1)	732 (9.2)	
Other (student, unpaid household work, retired)	285 (14.3)	281 (14.0)	304 (15.3)	198 (10.0)	1068 (13.4)	
Social class						<0.001
High	618 (30.9)	650 (32.5)	685 (34.4)	757 (38.1)	2710 (34.0)	
Medium	477 (23.9)	438 (21.9)	462 (23.2)	460 (23.1)	1837 (23.0)	
Low	820 (41.1)	833 (41.6)	725 (36.4)	687 (34.5)	3065 (38.4)	
Not classified/not working	82 (4.1)	81 (4.0)	118 (5.9)	85 (4.3)	366 (4.6)	

Notes: *p*-value: for qualitative variables: chi-square test; for quantitative variables: ANOVA.

**Table 2:** Vaccine hesitancy and refusal in adults. Population aged 18–64 years. Community of Madrid (Spain), 2019–2024.

	Vaccine Hesitancy		Vaccine Refusal	
	Crude PR (CI 95%)	aPR (CI 95%)	Crude PR (CI 95%)	aPR (CI 95%)
	Total		Total	
Year				
2019	1.00	1.00	1.00	1.00
2020	1.29 (0.96–1.73)	1.29 (0.96–1.73)	1.05 (0.69–1.59)	1.04 (0.68–1.57)
2021	2.18 (1.67–2.85) **	2.21 (1.69–2.88) **	0.38 (0.22–0.68) **	0.39 (0.22–0.68) **
2024	4.91 (3.86–6.25) **	5.04 (3.96–6.41) **	3.80 (2.72–5.31) **	4.00 (2.86–5.59) **
	Men		Men	
Year				
2019	1.00	1.00	1.00	1.00
2020	2.00 (1.21–3.31)	2.05 (1.24–3.34) **	1.40 (0.73–2.70)	1.40 (0.72–2.71)
2021	3.62 (2.28–5.76) **	3.76 (2.36–5.98) **	0.33 (0.12–0.91) *	0.34 (0.12–0.93) *
2024	7.83 (5.07–12.10) **	7.96 (5.15–12.31) **	4.73 (2.73–8.19) **	4.73 (2.71–8.27) **
	Women		Women	
Year				
2019	1.00	1.00	1.00	1.00
2020	1.00 (0.69–1.44)	0.97 (0.67–1.41)	0.85 (0.49–1.47)	0.83 (0.48–1.44)
2021	1.58 (1.13–2.21) **	1.59 (1.14–2.22) **	0.41 (0.21–0.83) *	0.42 (0.21–0.83) *
2024	3.71 (2.77–4.96) **	3.80 (2.84–5.09) **	3.30 (2.16–5.03) **	3.56 (2.34–5.41) **

Notes: PR: prevalence ratio. aPR: adjusted prevalence ratio by sex, age group, country of birth, education, and employment status.* *p* < 0.01; ** *p* < 0.01.

## Data Availability

The Directorate General of Public Health owns the data. Data availability can be consulted through the transparency portal: https://www.comunidad.madrid/transparencia/derecho-acceso-informacion-publica (accessed on 31 October 2025).

## Abbreviations

The following abbreviations are used in this manuscript:

aPR	Adjusted Prevalence Ratio
CI	Confidence Interval
CM	Community of Madrid
CATI	Computer-Assisted Telephone Interviewing
COVID-19	Coronavirus Disease 2019
HDI	Human Development Index
NCD	Noncommunicable Disease
SIVFRENT-A	Sistema de Vigilancia de Factores de Riesgo Asociados a Enfermedades No Transmisibles-población Adulta
SES	Socioeconomic Status
SD	Standard Deviation
WHO	World Health Organization
